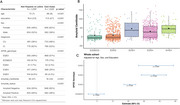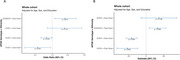# Apolipoprotein E and ethnicity interaction effects on amyloid‐PET status among East Asian and Not Hispanic and Latino White people

**DOI:** 10.1002/alz70856_107790

**Published:** 2026-01-09

**Authors:** Size Li, Qi Huang, Yihui Guan, Jing Zhang, Fang Xie

**Affiliations:** ^1^ Fudan University, Shanghai, Shanghai, China; ^2^ Huashan Hospital, Fudan University, Shanghai, shanghai, China; ^3^ Department of Nuclear Medicine and PET Center, Huashan Hospital, Fudan University, Shanghai, China; ^4^ Department of Nuclear Medicine & PET Center, Huashan Hospital, Fudan University, Shanghai, Shanghai, China

## Abstract

**Background:**

Apolipoprotein E (APOE) and ethnicity were proved to have strong effect on Alzheimer's disease. However, study on APOE effect on amyloid‐PET in East Asians was limited. Here, we assess the effects of APOE and race/ethnicity interaction effects on amyloid‐positivity and amyloid‐PET among East Asian and Not Hispanic and Latino White people.

**Method:**

Linear regression model was used to estimate the APOE and APOE/ethnicity interaction on amyloid‐PET among East Asians (*N* = 1529) and Not Hispanic and Latino White (ADNI, *N* = 1259). Logistic generalized estimating equations were used to estimate the APOE/ethnicity interaction effect on frequency of amyloid‐positivity (using cohort‐specific visual check). For estimation of APOE and APOE/ethnicity effect, the APOE ε3/ε3 was used as reference group. Both models were adjusted for age, sex and years of education.

**Result:**

APOE ε4 alleles were ascociated with higher risk amyloid deposition compared with ε3/ε3 group (ε2/ε4: β=37.68, *p* <0.001, ε3/ε4: β=20.96, *p* <0.001, ε4/ε4: β=36.27, *p* <0.001) among East Asians. However, ε2 alleles showed no protective effect on amyloid deposition in East Asians.

For APOE/ethnicity interaction effect (Figure 2), ε4 alleles in East Asians were associated with less amyloid deposition and amyloid positivity risk than Not Hispanic and Latino White population. In contrast, ε2 alleles were associated with higher risk of amyloid deposition and positivity in East Asians than Not Hispanic and Latino White people. Moreover, APOE ε2/ε4 showed similar effect between East Asians and White people on amyloid deposition and positivity.

**Conclusion:**

Through recent advances in AD‐related genetic cohorts, this study provided the largest‐to‐date overview of the association of APOE with amyloid‐PET risk in East Asians. APOE ε4 alleles were associated with less amyloid deposition risk and APOE ε2 alleles were associated with higher risk than White people. These novel insights are critical to guide AD clinical trial design and research.